# Genetic Variations in *SMAD7* Are Associated with Colorectal Cancer Risk in the Colon Cancer Family Registry

**DOI:** 10.1371/journal.pone.0060464

**Published:** 2013-04-03

**Authors:** Xuejuan Jiang, J. Esteban Castelao, David Vandenberg, Angel Carracedo, Carmen M. Redondo, David V. Conti, Jesus P. Paredes Cotoré, John D. Potter, Polly A. Newcomb, Michael N. Passarelli, Mark A. Jenkins, John L. Hopper, Steven Gallinger, Loic Le Marchand, María E. Martínez, Dennis J. Ahnen, John A. Baron, Noralane M. Lindor, Robert W. Haile, Manuela Gago-Dominguez

**Affiliations:** 1 Department of Preventive Medicine, University of Southern California, Los Angeles, California, United States of America; 2 Oncology and Genetics Unit, Complejo Hospitalario Universitario de Vigo, Servicio Galego de Saude (SERGAS), Vigo, Spain; 3 Genomic Medicine Group, Galician Foundation of Genomic Medicine, Complejo Hospitalario Universitario de Santiago, Servicio Galego de Saude (SERGAS), Instituto de Investigación Sanitaria de Santiago (IDIS), Santiago de Compostela, Spain; 4 Department of Surgery, University Hospital Santiago de Compostela, Santiago de Compostela, Spain; 5 Fred Hutchinson Cancer Research Center, Seattle, Washington, United States of America; 6 Centre for Molecular, Environmental, Genetic and Analytic Epidemiology, School of Population & Global Health, The University of Melbourne, Victoria, Australia; 7 Mount Sinai Hospital, University of Toronto, Toronto, Ontario, Canada; 8 University of Hawaii Cancer Research Center, Honolulu, Hawaii, United States of America; 9 University of California, San Diego Moores Cancer Center, San Diego, United States of America; 10 Denver VA Medical Center and University of Colorado, Denver, Colorado, United States of America; 11 Department of Medicine, University of North Carolina School of Medicine, Chapel Hill, North Carolina, United States of America; 12 Department of Health Science Research, Mayo Clinic, Scottsdale, Arizona, United States of America; Ohio State University Medical Center, United States of America

## Abstract

**Background:**

Recent genome-wide studies identified a risk locus for colorectal cancer at 18q21, which maps to the *SMAD7* gene. Our objective was to confirm the association between *SMAD7* SNPs and colorectal cancer risk in the multi-center Colon Cancer Family Registry.

**Materials and Methods:**

23 tagging SNPs in the *SMAD7* gene were genotyped among 1,592 population-based and 253 clinic-based families. The SNP-colorectal cancer associations were assessed in multivariable conditional logistic regression.

**Results:**

Among the population-based families, both SNPs rs12953717 (odds ratio, 1.29; 95% confidence interval, 1.12–1.49), and rs11874392 (odds ratio, 0.80; 95% confidence interval, 0.70–0.92) were associated with risk of colorectal cancer. These associations were similar among the population- and the clinic-based families, though they were significant only among the former. Marginally significant differences in the SNP-colorectal cancer associations were observed by use of nonsteroidal anti-inflammatory drugs, cigarette smoking, body mass index, and history of polyps.

**Conclusions:**

*SMAD7* SNPs were associated with colorectal cancer risk in the Colon Cancer Family Registry. There was evidence suggesting that the association between rs12953717 and colorectal cancer risk may be modified by factors such as smoking and use of nonsteroidal anti-inflammatory drugs.

## Introduction

It is estimated that inherited susceptibility contributes to ∼35% of all colorectal cancer (CRC) cases [1]. Recent progress through the application of genome-wide association studies (GWASs) have identified a number of common variants involved in the etiology of CRC[2]. Two GWAS [3], [4] identified a risk locus for CRC at 18q21, which maps to *SMAD7*, a functional candidate gene for CRC. Smad7 plays inhibitory roles in the transforming growth factor beta (TGF-β) signaling pathway [5], [6] which is involved in many cellular processes and has an important role in cancer development and progression [7]. Broderick et al. [3] identified three SNPs (rs4939827, rs12953717, rs4464148) in *SMAD7* associated with CRC and the SNP rs4939827 was later replicated as the top-ranking SNP at 18q21 by Tenesa et al. [4]. The association between rs4939827 and CRC risk was also confirmed in a recent meta-analysis [8]; however, significant between-study heterogeneity was observed. In addition, these susceptibility variants were found to be enriched in familial CRC [9], [10]. Furthermore, *SMAD7* expression was found to be lower in colorectal cancers than in adenomas regardless of 18q copy number status [11] and the risk allele at rs12953717 was significantly associated with lower *SMAD7* expression in lymphoblastoid cell lines [3], suggesting that allele-specific expression of *SMAD7* is likely to be the biological mechanism underlying the association between variations in 18q21 and CRC risk.

Given the SMAD7's role in the TGF-β signaling pathway [12] and the significant between-study heterogeneity in the reported association between SMAD7 SNPs and CRC risk, we looked to confirm the association between *SMAD7* SNPs and CRC risk in a large family-based case-control study based on the multi-center Colon Cancer Family Registry (Colon CFR), and to examine gene X environmental interactions to identify risk/protective factors that may affect the association between SMAD7 SNPs and CRC risk. Our case-unaffected sibling study design has been shown to be more powerful for controlling potential confounding from population stratification and detecting gene-environment interactions [13].

## Materials and Methods

### Study population

Data for this study were obtained through the Colon CFR, a National Cancer Institute (NCI)–funded registry of CRC cases, unaffected family members, and population-based controls. The registry is described in detail in Newcomb et al. [14] and Levine et al. [15]. Briefly, the Colon CFR is an international collaborative study initiated in 1997. Participants were recruited from six centers including centers in the University of Southern California Consortium (Arizona, Cleveland Clinic, Colorado, Dartmouth, Minnesota, North Carolina, and University of Southern California), Hawaii (Honolulu), Fred Hutchinson Cancer Research Center (Seattle, WA), Mayo Clinic (Rochester, MN), Cancer Care Ontario (Toronto, Canada), and University of Melbourne (Victoria, Australia) using population-based and clinic-based ascertainment strategies. Cases were recruited in two phases, from 1998 to 2002 (phase 1) and from 2002 to 2007 (phase 2). Phase 2 subjects were enriched in cases more likely to have a family history of CRC. All centers except Fred Hutchinson Cancer Research Center oversampled cases with multiple first-degree relatives reporting CRC or CRC cases diagnosed under age 50 to target families with excess CRC risk. First-degree and some second-degree relatives with CRC were also recruited from families with multiple CRC cases. The clinic-based sample represents multiple-case families at high risk of Hereditary Non-Polyposis Colorectal Cancer or other familial CRC phenotypes.

In the Colon CFR, population-based controls were only obtained from one of the Colon CFR sites (Fred Hutchinson Cancer Research Center), and the total sample size (N = 429) is much smaller than that of the sibling controls (N = 3,115). To make the most use of the available genetic data, in this investigation we used a case/unaffected sibling control design[13] with data from both population-based and clinic-based families in the main effect analyses. Cases were probands and siblings diagnosed with CRC and controls were siblings without CRC at the time of ascertainment. Diagnosis of CRC was based on the following six categories of confirmation [14]: pathologist review of slides; review of pathology report; cancer registry report or medical record(s) indicating treatment for the specific type of cancer; report on a death certificate; self-report; and report by a relative. Therefore, the unaffected status of the siblings was not established through colonoscopy. All cases were interviewed within 5 years of diagnosis (76% within 2 years). There were too few clinic-based case/control pairs for stratified analyses so all stratified analyses used the population-based families only. We excluded monozygous twins and subjects with unknown age or gender, and included only non-Hispanic white subjects. In addition, we also genotyped a random set of unrelated population-based controls (*n* = 429) from one of the Colon CFR sites (Fred Hutchinson Cancer Research Center). A total of 1,923 cases (1,640 population-based and 283 clinic-based) and their 3,115 unaffected sibling controls (2,621 population-based and 494 clinic-based) were included in the analyses.

### Ethics

All subjects signed an informed consent before providing data to the Colon CFR. Ethics approval for the study was obtained from the Institutional Review Boards at each CCFR site: University of Southern California Health Sciences Institutional Review Board, Mount Sinai Hospital Ethics Research Board, University of Hawaii Institutional Review Board, The University of Melbourne Central Human Research Ethics Committee, Fred Hutchinson Cancer Research Center Institutional Review Board, and Mayo Clinic Institutional Review Board.

### SNP Selection and genotyping

*SMAD7* was genotyped as part of an ongoing study of genes relevant to lipid peroxidation and apoptosis (5R01CA114472-02). Tagging single nucleotide polymorphisms (SNPs) were selected using the program Snagger20 [16] to cover all SNPs with a minor allele frequency (MAF) of ≥0.05 or greater with a pairwise r^2^ of ≥0.80 in the region covering each gene of interest as well as 20 kb upstream and 10 kb downstream of the gene. The linkage disequilibrium blocks were determined using data from the International HapMap Project White CEPH (Utah residents with ancestry from northern and western Europe) population (HapMap, release 21, July 2006; www.hapmap.org). Finally, three GWAS-identified SNPs, rs4939827, rs12953717, rs4464148, were also included. SNPs were genotyped on the Illumina GoldenGate platform (Illumina, Inc., San Diego, CA) [17] in the University of Southern California, Norris Comprehensive Cancer Center, Molecular Genomics Core facility, using DNA extracted from blood samples [14]. Quality control measures included testing for deviations from Hardy-Weinberg equilibrium (HWE) in non-Hispanic Whites, the inclusion of blinded interplate and intraplate replicates, and mixing cases and controls on genotyping plates. SNPs were excluded from the analysis if there were more than two errors on the replicate genotypes. Marker rs4939827 failed on the Illumina platform and was subsequently excluded. In this analysis, we report results for 23 tSNPs in *SMAD7*. A total of 133 blinded duplicate pairs were included for genotyping. Concordance for the duplicate samples was >99%.

### Microsatellite Instability Testing

All available tumors from the Colon CFR's Jeremy Jass Memorial Pathology Bank were assayed for instability at the following 10 microsatellites: BAT25, BAT26, BAT40, BAT34C4, D5S346, D17S250, D18S55, D10S197, ACTC, and MYCL as described previously [14]. Only subjects with clear results for at least four markers were included. Microsatellite Instability (MSI) data were available for 1,242 (64.4%) of cases (1,106 population-based and 136 clinic-based). Instability at >30% of the tested loci was defined as microsatellite instability high (MSI-H); instability at >10% of loci but <30% of loci was defined as microsatellite instability low (MSI-L); and those with instability at 0 loci were categorized as microsatellite stable (MSS).

### Tumor Location

Tumor location was obtained from the pathology report and was available for 1,778 (92.1%) of cases (1,566 population-based and 212 clinic-based). Right colon was defined as the cecum through the splenic flexure; left colon included the descending colon through the sigmoid colon; rectal tumors included the rectosigmoid junction and the rectum.

### Statistical Analysis

All statistical analyses were conducted using the R programming language and SAS v9.1 (SAS Institute Inc., Cary, NC).

MAF was estimated from the genotype data from unrelated population-based controls. Pairwise linkage disequilibrium between SNPs was estimated using the square of the correlation coefficient (*R* ^2^) and D-prime (D′) between markers. We also evaluated Hardy-Weinberg equilibrium for each SNP. No deviations were observed for all SNPs except rs1873190. Among the unrelated population-based controls, statistically significant reduced numbers of heterozygous genotypes of rs1873190 (155 observed vs. 186 expected under HWE; P exact = 0.0006) were observed. The genotyping data of this SNP showed clear genotype separation with a call rate of 98% and concordance rates of 100% among replicates.

In the analysis of main effects, the population- and clinic-based data were analyzed separately. We used multivariable conditional logistic regression with sibship as the matching factor and controlled for age (continuous) and sex. We assessed the SNP-CRC associations assuming a log-additive model. In all analyses, the lower frequency allele was coded as the “risk” allele and individuals were assigned a 0, 1, or 2 representing the number of risk alleles they possessed for that SNP. Test for haplotypic association was performed using SAS/Genetics and haplotype frequencies were estimated by the expectation–maximization algorithm.

Among the population-based families, we also examined the possibility that the SNP-CRC association was modified by other factors including gender, age, use of nonsteroidal anti-inflammatory drugs (NSAIDs), cigarette smoking, alcohol drinking, body mass index (BMI), physical activity (average weekly metabolic equivalent (MET) hours of physical activity throughout adulthood), history of diabetes, polyps, and ulcerative colitis, and family history of CRC in a first-degree relative as reported by the proband. All analyses within the exposure strata were specified in advance based on indications for potential effect modification in the literature. To test this hypothesis, dummy variables representing stratum-specific exposure were created for estimating stratum-specific results in one single conditional logistic regression model. P-values for interactions were estimated from likelihood ratio tests. We also evaluated differences in the association by tumor location (right, left, rectum/rectosigmoid junction) and MSI status (MSS, MSI-L and MSI-H) by stratifying the matched sets on the tumor characteristics of the case. We assigned the sets the MSI or tumor-site category of the case and included interaction terms in the conditional logistic regression models to estimate these stratum-specific odds ratios. Finally, we considered whether inclusion of cases recruited >2 years after diagnosis resulted in biased estimates and results were fundamentally unchanged after exclusion of these cases (data not shown).

## Results

Table 1 shows the demographic characteristics of the study population. A total of 1,854 sibships were included in this study. Among the participants, 1,640 cases and 2,621 controls were population-based and 283 cases and 494 controls were clinic-based. Data for tumor site and MSI status were available for 1,778 cases and 1,242 cases respectively.

**Table 1:** Selected characteristics of cases and sibling controls from participants in the Colon Cancer Family Registry.

	Population-based families (1, 592 sibships)			Clinic-based families (253 sibships)		
	Cases (N = 1,640)^1^	Sibling controls (N = 2,621)^1^	*P^4^*	Cases (N = 283)^1^	Sibling controls (N = 494)^1^	*P^4^*
**Personal characteristics**						
Mean age ±SD	53.6±10.8	54.0±11.7	*<0.001*	49.6±11.4	51.6±11.9	*<0.001*
Sex, no. (%)						
Male	838 (51.1)	1,167 (44.5)	*<0.001*	143 (50.5)	210 (42.5)	*0.039*
Female	802 (48.9)	1,454 (55.5)		141 (49.5)	284 (57.5)	
Center, no. (%)						
Ontario, Canada	298 (18.2)	494 (18.9)	-	0 (0)	0 (0)	-
USC Consortium, U.S.	336 (20.5)	461 (17.6)		42 (14.8)	50 (10.1)	
Melbourne, Australia	325 (19.8)	587 (22.4)		123 (43.5)	234 (47.4)	
Hawaii, U.S.	7 (0.4)	8 (0.3)		0 (0)	0 (0)	
Mayo Foundation, U.S.	271 (16.5)	516 (19.7)		118 (41.7)	210 (42.5)	
Seattle, U.S.	403 (24.6)	555 (21.2)		0 (0)	0 (0)	
BMI (kg/m^2^)^2^						
15–18 (underweight)	17 (1.0)	23 (0.9)	*0.049*	6 (2.1)	13 (2.6)	*0.53*
18–25 (normal)	572 (34.9)	1,042 (39.8)		99 (35.0)	181 (36.6)	
25–30 (overweight)	618 (37.7)	953 (36.4)		104 (36.8)	181 (36.6)	
30+ (obese)	375 (22.9)	539 (20.6)		61 (21.6)	97 (19.6)	
Unknown/missing	58 (3.5)	64 (2.4)		13 (4.6)	22 (4.5)	
Physical activity (MET hours)^3^						
0–6 (inactive)	391 (23.8)	604 (23.0)	*0.005*	42 (14.8)	100 (20.2)	*0.63*
6.1–20 (less active)	438 (26.7)	712(27.2)		55 (19.4)	119 (24.1)	
20.1–44 (active)	392 (23.9)	590 (22.5)		72 (25.4)	129 (26.1)	
44+ (very active)	345 (21.0)	565 (21.6)		112 (39.6)	141 (28.5)	
Unknown/missing	74 (4.5)	150 (5.7)		2 (0.7)	5 (1.0)	
Family history of CRC						
No first-degree relative	1,093 (66.7)	-		101 (35.7)	-	
≥1 first-degree relative	475 (29.0)	-		67 (23.7)	-	
Unknown/missing	72 (4.4)	-		115 (40.6)	-	
						
**Tumor characteristics**						
Site, no. (%)						
Right colon	546 (33.3)	-		87 (30.7)	-	
Left colon	477 (29.1)	-		44 (15.6)	-	
Rectum	543 (33.1)	-		81 (28.6)	-	
Unknown/missing	74 (4.5)	-		71 (25.1)	-	
MSI, no. (%)						
MSS	790 (48.2)	-		62 (21.9)	-	
MSI-L	141 (8.6)	-		15 (5.3)	-	
MSI-H	175 (10.7)	-		59 (20.9)	-	
Unknown/missing	534 (32.6)	-		147 (51.9)	-	

1 Non-Hispanic white family-based discordant siblings with age and sex data. ^2^Self-reported weight and height 2 years prior to questionnaire completion date used to calculate body mass index (BMI). ^3^Average weekly total lifetime MET hours. ^4^Estimated from 1-df likelihood ratio test from a conditional logistic regression model.

Results of the single SNP analysis are shown in Table 2. SNPs were sorted according to their position on chromosome 18. Assuming a log-additive model, a total of seven SNPs had a P value less than 0.05; after Bonferroni correction for the number of SNPs tested in the *SMAD7* gene, only two SNPs, rs11874392 and rs12953717, remained significantly associated with CRC risk among the population-based families. Each minor allele (T) of rs12953717 SNP was associated with a significantly increased risk of CRC (odds ratio-OR, 1.29; 95% confidence interval-CI, 1.12–1.49), whereas each minor allele (T) of rs11874392 was associated with a statistically significantly reduced risk of CRC (OR, 0.80; 95% CI, 0.70–0.92). These associations were similar among the population-based families and the clinic-based families, though they were only significant among the population-based families with its larger sample size. The two SNPs were highly correlated with each other (D′, 0.999; R^2^, 0.661). In a logistic regression analysis, the inclusion of rs11874392 did not significantly improve the fit of the model over that with rs12953717 alone (P = 0.88). Analysis of rs12953717 and rs11874392 revealed that CT and TA were the most common haplotypes (Table 3) and only TA was associated with higher risk of CRC (P = 9.0×10^−5^). Haplotype CA was not associated with CRC risk (P = 0.53). No significant associations were observed for the rs4464148 SNP or other SNPs evaluated in *SMAD7* after adjustment for multiple testing.

**Table 2:** *SMAD7* tagging SNPs and colorectal cancer risk in the Colon Cancer Family Registry.

	Location in	Major/minor			Population-based families		Clinic-based families	
Variant	*SMAD7*	allele	MAF^1^	Coordination	OR (95% CI)^2^	*P* ^2^	OR (95% CI)^2^	*P* ^2^
rs9945724	3′ UTR	C/T	0.06	44694487	0.89 (0.67–1.18)	*0.41*	0.70 (0.36–1.35)	*0.29*
rs17186485	intron	G/A	0.07	44703030	0.85 (0.66–1.10)	*0.22*	0.92 (0.49–1.70)	*0.78*
rs11874392	intron	A/T	0.49	44707154	0.80 (0.70–0.92)	*0.0016*	0.83 (0.60–1.14)	*0.24*
rs4939826	intron	C/A	0.08	44707404	0.91 (0.74–1.13)	*0.38*	0.91 (0.54–1.53)	*0.72*
rs12953717	intron	C/T	0.43	44707927	1.29 (1.12–1.49)	*0.0005*	1.31 (0.91–1.89)	*0.14*
rs884013	intron	C/T	0.05	44710128	0.97 (0.73–1.28)	*0.81*	1.01 (0.47–2.17)	*0.98*
rs12456328	intron	C/T	0.14	44711094	1.15 (0.95–1.40)	*0.15*	0.64 (0.41–1.01)	*0.054*
rs6507876	intron	T/G	0.09	44712919	0.94 (0.76–1.17)	*0.57*	0.98 (0.57–1.68)	*0.93*
rs4464148	intron	T/C	0.31	44713030	1.16 (1.00–1.34)	*0.049*	1.10 (0.76–1.60)	*0.61*
rs2337107	intron	C/T	0.40	44713321	1.13 (0.97–1.30)	*0.11*	1.20 (0.84–1.71)	*0.31*
rs7351039	intron	G/A	0.07	44714654	0.84 (0.63–1.14)	*0.26*	1.02 (0.53–1.94)	*0.96*
rs2337106	intron	G/C	0.47	44714901	0.94 (0.82–1.08)	*0.39*	1.05 (0.75–1.47)	*0.77*
rs7238442	intron	T/C	0.46	44715784	1.07 (0.93–1.22)	*0.34*	1.08 (0.78–1.48)	*0.65*
rs4939830	intron	G/A	0.09	44717056	1.16 (0.92–1.46)	*0.20*	0.73 (0.44–1.19)	*0.20*
rs4939832	intron	A/G	0.23	44719663	0.84 (0.71–0.99)	*0.037*	0.88 (0.59–1.30)	*0.51*
rs1873190	intron	G/A	0.33	44722354	0.90 (0.78–1.05)	*0.17*	1.17 (0.82–1.67)	*0.38*
rs3764482	intron	G/A	0.21	44722944	0.94 (0.79–1.11)	*0.47*	1.32 (0.87–1.99)	*0.19*
rs1316447	intron	G/A	0.18	44726674	1.20 (1.02–1.42)	*0.028*	0.89 (0.58–1.36)	*0.59*
rs3736242	intron	C/T	0.22	44728744	0.97 (0.83–1.14)	*0.73*	1.09 (0.74–1.63)	*0.66*
rs4939837	5′ UTR	G/A	0.35	44741996	0.86 (0.74–1.00)	*0.044*	0.92 (0.63–1.35)	*0.68*
rs7240215	5′ UTR	C/T	0.22	44744179	1.18 (1.01–1.39)	*0.037*	0.96 (0.63–1.46)	*0.84*
rs1867100	5′ UTR	C/G	0.14	44747964	1.09 (0.90–1.33)	*0.37*	1.17 (0.77–1.79)	*0.46*
rs7237225	5′ UTR	T/A	0.16	44752394	1.07 (0.87–1.32)	*0.50*	1.01 (0.61–1.67)	*0.97*

1 Minor allele frequencies (MAFs) were estimated using unrelated population-based controls. ^2^Results were estimated using a log-additive model, adjusted for age and sex.

**Table 3:** Colorectal cancer risk associated with haplotypes formed by SMAD7 SNPs rs12953717 and rs11874392.

Haplotype	Frequency in cases	Frequency in controls	OR (95% CI)^2^	*P* ^2^
CT	0.426	0.445	1.00	
TA	0.469	0.442	1.67 (1.29–2.16)	9.0×10^−5^
CA	0.098	0.102	1.14 (0.76–1.73)	0.53
TT	0.00003	0.0002	-	-

Among the population-based families, we assessed the association between rs12953717 and CRC risk after stratification by related risk/protective factors (Table 4). We observed modest and marginally significant differences in the disease association by use of NSAIDs, cigarette smoking, BMI and history of polyps. The strongest associations were observed among current NSAID users, non-/former smokers, overweight individuals (25≤BMI<30) and individuals with a prior diagnosis of polyps, respectively. There were no statistically significant differences in the SNP-CRC association according to gender, age, alcohol drinking, physical activity, diabetes, and ulcerative colitis. The effect of rs12953717 seemed to be stronger among individuals without a family history of CRC than among those with a family history; however, this difference was not statistically significant (P = 0.39). When the association between rs11874392 and risk of CRC was examined, similar but less pronounced differences by the above factors were observed.

**Table 4:** Stratified analyses of rs12953717 and rs11874392 by known risk/protective factors among population-based families.

	Risk associated with T allele of rs12953717			Risk associated with T allele of rs11874392		
	OR (95% CI)^1^	*P_Walds_* 1	*P_Interaction_* ^2^	OR (95% CI)^1^	*P_Walds_* 1	*P_Interaction_* ^2^
**Gender**						
Men	1.28 (1.07–1.54)	*0.0073*	*0.91*	0.82 (0.69–0.98)	0.026	*0.66*
Women	1.30 (1.09–1.55)	*0.0034*		0.79 (0.67–0.93)	0.0041	
**Age**						
<55	1.22 (1.01–1.46)	*0.039*	*0.33*	0.83 (0.70–0.98)	0.031	*0.66*
≥55	1.38 (1.13–1.69)	*0.0019*		0.78 (0.65–0.95)	0.014	
**NSAIDs**						
Nonusers	1.30 (1.09–1.55)	*0.0029*	*0.073*	0.77 (0.66–0.91)	0.0017	*0.34*
Former users	1.06 (0.83–1.35)	*0.63*		0.92 (0.73–1.15)	0.47	
Current users	1.53 (1.19–1.98)	*0.0011*		0.77 (0.60–0.97)	0.027	
**Cigarette smoking**						
Nonsmokers	1.37 (1.13–1.66)	*0.0014*	*0.050*	0.78 (0.65–0.93)	0.0066	*0.26*
Former smokers	1.36 (1.12–1.65)	*0.0019*		0.77 (0.64–0.92)	0.0043	
Current smokers	0.89 (0.64–1.24)	*0.50*		1.00 (0.74–1.36)	0.98	
**Alcohol drinking**						
Nondrinkers	1.35 (1.06–1.73)	*0.014*	*0.78*	0.79 (0.63–0.98)	0.033	*0.76*
Former drinkers	1.33 (0.99–1.79)	*0.057*		0.74 (0.57–0.96)	0.021	
Current drinkers	1.24 (1.04–1.48)	*0.016*		0.82 (0.69–0.96)	0.016	
**BMI**						
<25	1.19 (0.97–1.46)	*0.098*	*0.053*	0.82 (0.68–0.99)	0.043	*0.39*
25-<30	1.51 (1.23–1.86)	*0.00008*		0.74 (0.61–0.89)	0.0017	
≥30	1.09 (0.83–1.42)	*0.53*		0.89 (0.70–1.13)	0.33	
**Physical activity**						
Inactive or less active	1.23 (1.02–1.48)	*0.030*	*0.73*	0.77 (0.65–0.92)	0.0036	*0.30*
Active or very active	1.28 (1.06–1.55)	*0.011*		0.86 (0.72–1.02)	0.090	
**Diabetes**						
No	1.27 (1.09–1.47)	*0.0017*	*0.52*	0.80 (0.70–0.92)	0.0020	*0.95*
Yes	1.45 (0.97–2.15)	*0.068*		0.81 (0.56–1.17)	0.26	
**Polyps**						
No	1.13 (0.95–1.36)	*0.18*	*0.087*	0.89 (0.75–1.05)	0.16	*0.22*
Yes	1.39 (1.13–1.72)	*0.0020*		0.77 (0.64–0.94)	0.0098	
Ulcerative Colitis						
No	1.26 (1.08–1.46)	*0.0025*	*0.68*	0.81 (0.70–0.93)	0.0025	*0.83*
Yes	1.48 (0.68–3.23)	*0.32*		0.87 (0.44–1.72)	0.69	
**Family history of CRC**						
No first-degree relative	1.35 (1.13–1.61)	*0.00077*	*0.39*	0.75 (0.64–0.89)	0.00075	*0.21*
≥1 first-degree relative	1.18 (0.90–1.56)	*0.23*		0.91 (0.71–1.16)	0.45	

1 Adjusted for age and sex. ^2^P for interaction was estimated from likelihood-ratio test.

We also examined the possible heterogeneity of the SNP effect by tumor location and MSI status (Table 5). The strongest associations were observed for distal colon cancer and MSI-L/MSI-H tumors than for other tumors; however, neither difference was statistically significant.

**Table 5:** SNP rs12953713 and rs11874392 by tumor characteristics among population-based families.

	Risk associated with T allele of rs12953717			Risk associated with T allele of rs11874392		
	OR (95% CI)^1^	*P_Walds_* 1	*P_Interaction_* ^2^	OR (95% CI)^1^	*P_Walds_* 1	*P_Interaction_* ^2^
**Tumor location**						
Proximal colon	1.24 (0.96–1.59)	*0.10*	*0.37*	0.84 (0.67–1.05)	0.13	*0.43*
Distal colon	1.49 (1.13–1.95)	0.0041		0.70 (0.54–0.90)	0.0052	
Rectum	1.14 (0.90–1.46)	*0.28*		0.86 (0.67–1.09)	0.22	
**MSI status**						
MSS	1.21 (0.98–1.49)	*0.073*	*0.61*	0.86 (0.71–1.05)	0.13	*0.44*
MSI-L	1.52 (0.95–2.44)	*0.080*		0.69 (0.43–1.10)	0.12	
MSI-H	1.41 (0.90–2.20)	*0.13*		0.67 (0.45–1.00)	0.049	

1 Adjusted for age and sex. ^2^P for interaction was estimated from 2 degrees of freedom likelihood-ratio test of the interaction term between the tumor characteristics and SNP rs12953713.

To address potential survival bias due to the inclusion of cases interviewed up to 5 years after diagnosis, we repeated the analyses of main effects including only probands interviewed within 2 years of diagnosis and their unaffected siblings and results were fundamentally unchanged (data not shown).

## Discussion

Consistent with the two GWAS [3], [4], we found a statistically significant association between two *SMAD7* polymorphisms, rs12953717 and rs11874392, and CRC in this large family-based case-control study. We observed suggestions that the association of rs12953717 and CRC risk may be modified by use of NSAIDs, cigarette smoking, BMI and history of polyps.

The underlying causal variant for the association between 18q21 variation and CRC risk remains unknown. Pittman et al. [11] reported that a C to G SNP at 44,703,563 bp, a SNP in strong linkage disequilibrium with other genetic variations around 18q21, may be the functional variant responsible for 18q21-associated variations in CRC risk through differential *SMAD7* expression and subsequent TGF-β signaling. Compared to the C allele, the risk allele G forms weaker protein–DNA complexes with nuclear extracts and was associated with a reduced expression of *SMAD7* in the colorectum. However, it remains unclear how such alterations may promote carcinogenesis. It has been suggested that *SMAD7* may induce tumorigenicity by blocking TGF-β-induced growth inhibition and apoptosis [18]. The expression of *SMAD7* is very low in epithelial tissues, but is up-regulated in several cancers. Over-expression of *SMAD7* has been shown to inhibit TGF-β-mediated induction of endogenous heme oxygenase-1 (*HO-1*) gene expression [19], an adaptive defense against oxidant stress [20]. In colon cancer cells, stable expression of *SMAD7* blocks the TGF-β-mediated activation of *NFκB* [5], a critical molecule in oxidative-stress-induced apoptosis. In addition, CRC patients with deletion of *SMAD7* were found to have a favorable clinical outcome [21]. Conversely, amplification of this gene was associated with a significantly worse prognosis, with a graded effect depending on *SMAD7* gene copy number [21].

Given that *SMAD7* is involved in intestinal inflammation through its regulation of TGF-β signaling [22], [23] the association between *SMAD7* SNPs and CRC risk may be modified by factors affecting inflammation. In human inflammatory bowel tissues, TGF-β1 signaling is disrupted by the up-regulation of *SMAD7*, leading to an enhanced production of inflammatory cytokines. Inhibition of *SMAD7* with a specific antisense oligonucleotide can restore the immunosuppressive TGF-β1 signaling and suppress the inflammatory cytokine production [23]. In our study, there is some indication of differences in the association between rs12953717 and CRC risk by NSAID use, with the effect of the SNP being most pronounced among current users and least among former users. In a population-based case-control study of colon cancer, Slattery et al. [24] also observed slightly stronger associations of rs4939827 and rs12953717 for individuals reporting recent aspirin/NSAID use. Our findings are also consistent with those from a recent study suggesting that CRC-specific survival in postmenopausal women varied according to *SMAD7* genotype (rs4939827 and rs4464148) among patients who were regularly using NSAIDs before diagnosis, but not among never-users and former users of NSAIDs [25]. Studies have shown that disruption of TGF-β1 signaling due to high levels of *SMAD7* is a feature of colitis and blocking *SMAD7* restores TGF- β1 signaling in colitis [26]. Since non-/former smokers have higher risk of ulcerative colitis compared with current smokers [27], our observation of a stronger *SMAD7* SNP association among non-/former smokers would lend some support to the inflammation hypothesis. We consistently observed a higher CRC OR for rs12953717 among individuals with ulcerative colitis than those without, although this difference did not reach statistical significance, possibly due to the small number of individuals with ulcerative colitis. On the other hand, smoking increases the risk of the other form of IBD, Crohn's disease, but we were unable to examine the SNP-CRC association stratified by history of Crohn's disease due to the small number of individuals with this disease (N = 32). In addition, the observation of a BMI interaction could also suggest the involvement of inflammation pathways, since obesity is associated with a state of chronic inflammation [28]. However, the observation of the effect of SMAD7 genetic variants only among overweight individuals but not among obese individuals was unexpected and requires confirmation.

*SMAD7* may also function through insulin-related pathways. SMAD7 protein expression in the renal cortex is decreased in diabetes [29] and conditional expression of *SMAD7* in pancreatic β cells disrupts TGF-β signaling and induces reversible diabetes mellitus [30]. In addition, the *SMAD7* SNP rs3764482 (IVS2 –21C>T) has been associated with a reduced risk of type 2 diabetes in mice [31]. We explored this possible mechanism by examining differences in the association of the SNP with CRC risk by diabetes status but did not find strong evidence for an interaction with diabetes (Table 3). The association between rs12953717 and CRC risk remained among non-diabetic individuals.

In addition to confirming the associations between *SMAD7* SNPs and CRC risk, we further defined these associations according to other known risk/protective factors for CRC and tumor characteristics. We found a stronger SNP association among individuals with a prior history of polyps. Further exploration by the type, number and size of polyps was not performed because detailed information on these characteristics is lacking. The exact biological mechanism for the interaction between polyps and *SMAD7* is unknown; however, a recent study in prostate cancer cells found that *SMAD7* can interact with adenomatous polyposis coli (APC) protein in linking the TGF-β type I receptors to the microtubule system to promote cell migration [32], indicating a possible role of *SMAD7* in the progression from adenomatous polyps to CRCs. Interpretation of this result needs to be cautious, because the proportion of subjects who had been examined for polyps prior to cancer diagnosis is unknown. There was some indication of differences in the effect of *SMAD7* SNPs by tumor location. We observed a significant association of rs12953717 for distal colon tumors, but not for proximal colon tumors and rectal tumors. Such finding is consistent with Curtin et al. [33], who also reported significant associations for distal colon tumors for two *SMAD7* SNPs but not for proximal colon and rectal tumors. Similarly, Slattery et al. [24] observed slightly stronger associations for distal than for proximal colon cancers. However, no significant difference in the effect size of rs4939827 by tumor site was reported by Broderick et al. [3]. On the contrary, Tenesa et al. [4] found that rs4939827 was more strongly associated with risk of rectal cancer than for colon cancer. Consistent with most prior studies [4], [24], [34], we did not find any significant differences in the SNP associations by age, gender, family history and MSI status.

Our study has a number of limitations. First, SNP rs4939827 was excluded from our analysis due to genotyping failure; however, given the perfect linkage disequilibrium (R^2^ = 1.0 in HapMap CEU data Release 28.) between rs4939827 and rs11874392, there is unlikely to have been any loss of information due to this exclusion. Second, the Bonferroni method to adjust for multiple comparisons may be so conservative that some potentially important polymorphisms, such as the variant identified in a previous GWAS of CRC rs4464148 [3], may be overlooked. On the other hand, the observed weaker associations of other SMAD7 SNPs (rs4939832, rs1316447, rs4939837, rs7240215) with CRC risk have not been widely replicated by other studies and therefore could have been chance findings. Third, we used a case-unaffected sibling study design that may reduce the power to detect association between genetic variations and CRC risk because of overmatching on genotypes between cases and their unaffected siblings [13]; however, such design does not lead to biases in estimating genetic relative risks, and is more powerful for detecting gene-environment interactions and controls for potential confounding from population stratification. And lastly, we did not observe statistically significant associations between these common *SMAD7* SNPs and CRC risk in the clinic-based families, possibly due to the limited sample size in the clinic-based dataset. Alternatively, the genetic predisposition in the multiple-case, clinic-based families may be largely due to other yet-to-be-identified genetic variations with stronger effects.

In summary, using data from the Colorectal Cancer Family Registry, we confirmed the association between *SMAD7* and CRC found in GWAS. Further studies are needed to confirm our results stratified by demographic factors and tumor characteristics and to elucidate the relevant biological mechanisms. With the growing epidemiological evidence linking *SMAD7* to CRC susceptibility, studies are needed to investigate potential biological mechanisms by which *SMAD7* contributes to the development of CRC.
